# The MHC Class II Transactivator CIITA: Not (Quite) the Odd-One-Out Anymore among NLR Proteins

**DOI:** 10.3390/ijms22031074

**Published:** 2021-01-22

**Authors:** Jorge Alfonso León Machado, Viktor Steimle

**Affiliations:** Département de Biologie, Université de Sherbrooke, 2500 Boul., Sherbrooke, QC J1K 2R1, Canada; jorge.alfonso.leon.machado@usherbrooke.ca

**Keywords:** CIITA, NLRC5, MHC genes, gene regulation

## Abstract

In this review, we discuss the major histocompatibility complex (MHC) class II transactivator (CIITA), which is the master regulator of MHC class II gene expression. CIITA is the founding member of the mammalian nucleotide-binding and leucine-rich-repeat (NLR) protein family but stood apart for a long time as the only transcriptional regulator. More recently, it was found that its closest homolog, NLRC5 (NLR protein caspase activation and recruitment domain (CARD)-containing 5), is a regulator of MHC-I gene expression. Both act as non-DNA-binding activators through multiple protein–protein interactions with an MHC enhanceosome complex that binds cooperatively to a highly conserved combinatorial cis-acting module. Thus, the regulation of MHC-II expression is regulated largely through the differential expression of CIITA. In addition to the well-defined role of CIITA in MHC-II GENE regulation, we will discuss several other aspects of CIITA functions, such as its role in cancer, its role as a viral restriction element contributing to intrinsic immunity, and lastly, its very recently discovered role as an inhibitor of Ebola and SARS-Cov-2 virus replication. We will briefly touch upon the recently discovered role of NLRP3 as a transcriptional regulator, which suggests that transcriptional regulation is, after all, not such an unusual feature for NLR proteins.

## 1. Introduction

The major histocompatibility complex (MHC; in humans, human leukocyte antigen, HLA) class I and class II molecules are antigen-presenting molecules for CD8+, and CD4+ T cells, respectively, and are therefore of crucial importance for the cellular immune response. Their expression is mainly controlled at the level of gene transcription. MHC-II genes show a highly controlled developmental, cell-type and stimulus-specific expression with constitutive expression confined to professional antigen-presenting cells (APCs) such as dendritic cells (DCs), B cells, macrophages, and thymic epithelial cells. Other cell types are negative; however, the expression can be induced in diverse cell types by different stimuli, most prominently IFNγ. The study of a rare severe combined primary immunodeficiency called the bare lymphocyte syndrome (BLS) led to the identification of class II transactivator (CIITA) and of the three genes coding for the subunits of the heterotrimeric RFX (regulatory factor X-box) complex binding to the X-box in the highly conserved W/SXY module found in all MHC-II gene promoters. CIITA has been recognized as the “master regulator” of MHC-II expression since the differential expression of MHC-II genes is largely due to the differential expression of CIITA. CIITA is a founding member of the NLR (nucleotide-binding and leucine-rich-repeat-containing) protein family, but for a long time, it stood apart in this protein family as the only transcriptional regulator. MHC-I genes, on the other hand, are expressed ubiquitously in nucleated cells; however, their expression levels differ widely, and they also show inducible expression, e.g., by IFNγ. In addition to other regulatory elements, the MHC-I gene promoter also contains a functional W/SXY element (also called site α), and CIITA was found to contribute to MHC-I activation under certain conditions. However, it was also clear that many cell types express high levels of MHC-I in the complete absence of CIITA. This conundrum was resolved when it was recognized that the closest homolog of CIITA, NLRC5, functions as an MHC-I activator. In this review, we will mainly focus on CIITA but draw parallels to NLRC5 where appropriate.

## 2. Identification of CIITA, the Master Regulator of MHC-II Expression

CIITA was identified through a genetic complementation approach in the HLA-II-negative cell line RJ2.2.5, which was derived from the Burkitt lymphoma line Raji by γ-irradiation followed by negative immunoselection [1,2]. The 4.5 kb cDNA coded for an 1130 amino acid protein, which was later identified as isoform III of CIITA (see below). CIITA mutations were identified in RJ2.2.5 and the B cell line BLS-2 from BLS complementation group A [2,3].

After the identification of CIITA, it was rapidly established that CIITA also controls IFNγ-induced MHC-II expression [4,5]. CIITA mRNA is induced by IFNγ, and ectopic expression of CIITA is necessary and sufficient to induce MHC-II gene expression [4,5]. This has since been repeated in many different cell lines. CIITA controls MHC-II expression quantitatively, and there is a close correlation between levels of CIITA and MHC-II mRNA expression levels in different tissues [6].

## 3. CIITA, a Founding Member of Mammalian NLR Proteins

CIITA is the founding member of the NLR protein family [7]. The nucleotide-binding domain also has been described as the NACHT (NAIP, CIITA, HET-E, TP1) domain and the protein family as the CATERPILLER (caspase activation and recruitment domain (CARD), transcription enhancer, R (purine)-binding, pyrin, lots of leucine repeats) family [8,9,10]. CIITA was, however, for a very long time, the most exceptional member of this protein family in that it was the only transcriptional regulator.

**Figure 1:**
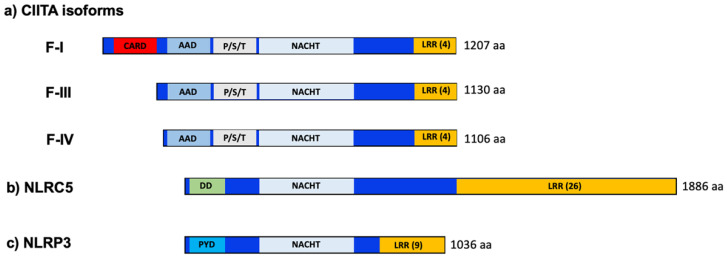
Domain structure of nucleotide-binding and leucine-rich-repeat (NLR) proteins CIITA (**a**), NLRC5 (**b**) and NLRP3 (**c**). Abbreviations: AAD, acidic activation domain; CARD, caspase activation and recruitment domain; DD, death domain; F-I, F-III, F-IV, isoforms-I, -III and -IV; LRR, leucine-rich repeats; NACHT, NAIP, CIITA, HET-E, TP1 domain; P/S/T, proline-serine-threonine rich domain; PYD, pyrin domain.

The CIITA protein structure is characterized by an N-terminal acidic domain, a region rich in prolines, serines, and threonines (P/S/T domain), a central nucleotide-binding domain (GTP domain) and at least four C-terminal leucine-rich repeats (LRRs; Figure 1).

Three alternative promoters (pI, pIII, and pIV) and corresponding exons one generate three different isoforms of CIITA (isoforms I, III, and IV) differing in their N-termini. Isoform IV is initiated by an AUG in the common exon two, whereas isoforms I and III carry their own initiator AUGs leading to N-terminal extensions of 101 and 24 amino acids, respectively. The N-terminal extension of isoform I shows homology to caspase activation and recruitment domain (CARD) and was found to increase MHC-II transcription somewhat [11] (Figure 1).

While there is a close correlation between CIITA and MHC-II expression in many cell lines and tissues, there have nevertheless been many post-translational modifications described for CIITA. Modifications include phosphorylation, acetylation and deacetylation, and ubiquitination (reviewed in [12]). Furthermore, CIITA has been shown to self-associate via its GTP- and LRR-domains and also to oligomerize, contributing to nuclear localization (see also below) and transactivation [13,14,15,16].

CIITA has never been shown to bind directly to DNA and has therefore frequently been described as a coactivator, although the term is somewhat misleading since it is the principal activator of MHC-II expression. The N-terminal acidic domain of CIITA has been shown to function as a potent transactivation domain and shares many functionalities with the acidic domain of the viral activator VP16 [17,18,19,20]. CIITA activates transcription initiation and elongation through multiple mechanisms (reviewed in [21,22]). CIITA recruits components of the general transcription machinery, such as TFIID and TFIIB, it induces phosphorylation of RNA polymerase II and interacts with *P*-TEFb. It also recruits chromatin remodeling coactivators such as p300, CBP, PCAF, and BRG1 [21,22]. CIITA also has been shown to interact with the 19S proteasomal subunit Sug1 [23,24]. CIITA also has been reported to have histone acetylase (HAT) activity, to possess an endogenous kinase activity, and can functionally replace the TFIID component TAF1 (reviewed in [25]) (Table 1).

Nucleo-cytoplasmic transport and localization of CIITA is a very complex subject with many different mutations distributed over the whole length of the protein affecting subcellular localization and transport. Both nuclear localization signals and nuclear export signals have been described, which may explain that wild-type CIITA shows a close to 50:50 distribution between nucleus and cytoplasm [15,26,27,28,29,30,31,32]. Several signals depend on post-translational modifications, such as the PCAF-dependent acetylation of lysines in the acidic domain [33]. Deletion of the acidic and P/S/T domains leads to an exclusively cytoplasmic CIITA fragment (L335), which can, however, be driven very efficiently to an exclusively nuclear localization by the addition of an SV40 large T nuclear localization signal (NLS-L335). Interestingly, both NLS-L335 and L335 are strongly dominant-negative, suggesting that essential interactions with protein partners can occur both in the nucleus and in the cytoplasm [31,34].

CIITA is a short-lived protein, with isoform III showing a half-life of about 30–45 min in pulse-chase experiments [35]. Degradation signals reside in the N-terminal P/S/T and acidic domains and in the extreme N-terminus itself [35,36]. N-terminal epitope-tags stabilize the protein, and the first 10 amino acids of CIITA isoform III can destabilize a heterologous protein [36]. Interestingly, the transactivation potential is inversely correlated to stability, and CIITA isoform III interacts more efficiently with the RFX complex and the transcription machinery when compared to a truncated version [36]. Mono-ubiquitination was shown to increase CIITA-dependent MHC-II transactivation in a non-degradative manner [37].

## 4. Identification of NLRC5 as an MHC-I Regulator

NLRC5 was identified as an MHC-I regulator (also referred to as CITA; class I transactivator) through a knockdown approach by Kobayashi and colleagues [38]. This activity was mediated by the W/SXY motif and the MHC enhanceosome in the MHC-I promoters [39,40]. NLRC5 and CIITA are the closest homologs among NLR proteins with respect to their GTP and LRR domains, but NLRC5 differs strongly from CIITA in the N-terminal domain [38,41]. There is no P/S/T region or acidic activation domain in NLRC5, but it rather carries N-terminal to the GTP domain a region with weak homology to a CARD, which can potentially adopt a related death domain (DD) fold (Figure 1) ref. [42].

Domain swap experiments yielded very interesting information concerning the function of the NLRC5 DD [43]. NLRC5 exclusively activated an MHC-I reporter construct, whereas CIITA showed a strong preference for MHC-II in HEK293T cells [43]. It had been shown earlier that N-terminal deletions of either the acidic (∆-163) or the acidic and the P/S/T domain (L335) of CIITA lead to strong dominant-negative phenotypes [31,34]. Surprisingly, the fusion of the DD to ∆-163 or to L335 led to constructs that were very efficient in activating both MHC-I and MHC-II genes [43]. This showed first that the DD could act as a bona fide transcriptional activation domain, and second, it demonstrated that the specificity for MHC-I or MHC-II promoters resulted from a combined action of the activation domains and the GTP- and LRR-domains. Finally, it revealed that under certain conditions, CIITA could be very efficiently recruited to MHC-I promoters.

NLRC5-deficient mice confirmed its importance for MHC-I expression, at least in certain cell subsets. While MHC-I expression was only mildly affected in APCs, such as macrophages, there was a strongly reduced MHC-I expression in lymphocytes such as T, NKT and NK cells [41,44,45,46].

## 5. Cis-Acting Elements, MHC Enhanceosome, CIITA- and NLRC5-Cistromes

**Figure 2:**
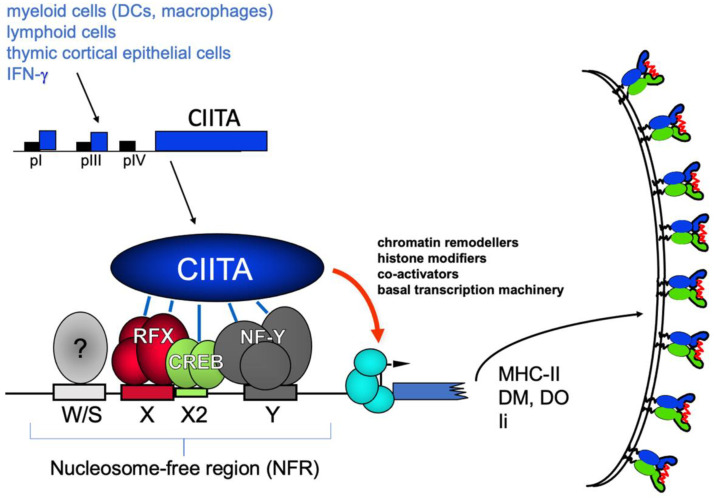
The master regulator CIITA and the major histocompatibility complex (MHC)-II enhanceosome.

The MHC-II promoter is the prototype of a composite regulatory element, which is often referred to as the W/SXY or SXY module. It is composed of four cis-acting elements, the W/S, X (or X1), X2 and Y boxes, which are present in classical and non-classical MHC-II gene promoters in a fixed order, orientation and spacing. The W/S element is bound by an as yet unknown factor, but the other three binding complexes have been well defined. The X-box is bound by the heterotrimeric RFX (regulatory factor X) complex composed of the DNA-binding subunit RFX5, RFX-AP (RFX-associated protein) and RFX-ANK or RFX-B (RFX-ankyrin-containing protein or RFX-defective in BLS group B). The corresponding genes are mutated in BLS complementation groups C, D and B, respectively [47,48,49,50]. The X2-element is bound by the cyclic-AMP-responsive-element-binding protein (CREB) [51], whereas the Y-box, which is an inverted CCAAT-element, is bound by the major CCAAT-box-binding protein complex NF-Y [52,53]. These protein complexes bind cooperatively to the W/SXY module in vitro and in vivo and form a platform in the manner of an enhanceosome, to which CIITA is recruited through multiple protein–protein interactions [30,54,55,56,57]. Surprisingly, the W/SXY-binding complex is completely inert with respect to MHC-II activation in vivo, and it is only the recruitment of CIITA to the complex which is both necessary and sufficient to induce MHC-II expression (Figure 2).

Recently, the CCR4-NOT (carbon catabolite repressor 4-negative on TATA) complex has been implicated in the non-expression of MHC-II genes [58]. Knockdown or knock-out of CCR4-NOT subunits led to the expression of MHC-II genes in the absence of CIITA [58]. This suggests that the CCR4-NOT complex is necessary for the tightness of the regulation of MHC-II genes and to prevent leaky expression from MHC-II promoters in the absence of CIITA. CIITA must then, either directly or indirectly, relieve the suppression of CCR4-NOT to activate MHC-II transcription. How this is achieved mechanistically is unknown at present.

CIITA induces transcription-associated histone modifications over the MHC-II promoter in two sequential phases, the first of which is characterized by a rapid increase in histone H4 acetylation over a large region. The second phase coincides with transcription and is restricted to short upstream regions, where histone acetylation and methylation occurs. This second phase is dependent on RNA pol-II-mediated transcription elongation [59]. Induction of CIITA by IFNγ leads to the eviction of nucleosomes over the MHC-II promoters and the appearance of a nucleosome-free region (NFR) [60] (Figure 2).

The W/SXY module is found in the proximal promoters of all classical MHC-II genes (HLA-DR, -DQ and -DP) and also in several associated genes such as the invariant chain (Ii), HLA-DM and HLA-DO. In addition to these proximal promoter elements, more distal regulatory elements were identified throughout the MHC-II locus on human chromosome 6. These distal regulatory elements were identified based on their homology to either the MHC-II X-box (termed XL elements) or to the W/SXY motif. These elements bind RFX and CIITA in vivo and function as enhancers of MHC-II expression [61,62]. These elements are involved in long-distance interactions and help to achieve a coordinated and specific expression of MHC-II genes. Interestingly, several of these elements are bound by the CCCTC-binding factor CTCF, and knockdown of CTCF leads to a reduction of MHC-II gene expression concomitantly with a reduction in long-distance interactions [22,63,64].

Recently, the region between the HLA-DRB1 and HLA-DQA1 loci in the MHC-II region was shown to display characteristics of a super-enhancer (SE) region [65,66]. Two SEs were identified in this region and termed DR/DQ-SE and XL9-SE, and were shown to be highly enriched in disease-associated SNPs. CRISPR/Cas9-mediated deletion of the DR/DQ-SE led to a reduction of HLA-DRB1 and HLA-DQA1 expression. Local chromatin interactions were disrupted by SE-deletions, suggesting that the SEs are involved in the organization of the local three-dimensional chromatin architecture [66].

Interestingly, the MHC-I gene promoters also possess a W/SXY module, which also has been described as site α. However, the pattern of expression of MHC-I genes is only partly overlapping with that of MHC-II genes, and thus it is not surprising that MHC-I promoters also possess other regulatory elements implicated in constitutive and inducible expression. MHC-I genes are expressed ubiquitously, but their expression levels vary greatly with high levels of expression in hematopoietic cells. Furthermore, their expression can be modulated by different stimuli, prominently among them IFNγ. Somewhat surprisingly, it was found that CIITA can activate MHC-I expression, especially in cell lines that were very low or negative for MHC-I expression [67,68]. Expression was dependent on the presence of site α containing the W/SXY module and recruitment of the RFX complex [67,68,69]. On the other hand, CIITA KO mice and cells from BLS group A did not show a notable reduction in MHC-I expression and many cell lines express high levels of MHC-I in the complete absence of CIITA. This puzzle was at least partially resolved when NLRC5 was identified as an MHC-I activator and was shown to be recruited to the W/SXY module in the MHC-I promoters [38,39,40].

Analysis of the CIITA cistrome led to some very interesting results. A genome-wide scan for promoter-proximal-binding of CIITA via a ChIP-chip (chromatin IP-microarray) analysis yielded only nine validated targets in addition to MHC genes [70]. ChIP-seq analysis led to somewhat higher numbers of in vivo-binding sites with 843 or 480 hits, respectively [71,72]. However, this is still a very limited number of binding sites when compared to other transcription regulators that often show thousands of binding sites in the genome. Only a few novel genes in addition to MHC-II genes were found to be regulated by CIITA, and computational analysis showed that CIITA bound mostly through XY box motifs in one analysis [72], although the other study found that 60% of CIITA-binding sites were not associated with RFX5 binding [71]. The most astonishing results were, however, obtained in an NLRC5 ChIP-seq analysis, where only a highly restricted set of 12 targets in classical and non-classical MHC-I genes was identified [73]. NLRC5 and CIITA were found to have nonredundant target sets, although both were dependent on a W/SXY cis-acting module. Bioinformatic analysis revealed that the W/S element was essential in conferring the NLRC5 specificity [73]. The extremely restricted target gene set of NLRC5 is highly unusual and possibly unique among transcriptional regulators so far and illustrates the exquisite specificity that can be achieved through combinatorial cis-acting elements and cooperatively binding trans-acting factors.

## 6. Regulation of CIITA Gene Expression

As described above, the regulation of MHC-II gene expression is largely regulated through CIITA and levels of MHC-II, and CIITA mRNA expression correlate closely in various murine tissues and cell lines [6]. Thus, differential MHC-II expression is largely due to differential CIITA expression. Expression of CIITA appears to be mostly regulated at the level of transcription, although diverse post-transcriptional modifications of CIITA have also been described. Regulation of CIITA gene expression has been reviewed in some detail, and we will here sum up the most salient features, mainly based on studies of CIITA promoter knock-out mice [21,74]. Knock-out of promoter IV (pIV) of CIITA showed that this promoter was essential for the IFNγ-induced expression of CIITA in non-hematopoietic cells [75]. Macrophages, on the other hand, were shown to display normal IFNγ-induced CIITA and MHC-II expression in pIV KO mice [75]. A surprising finding was that pIV is essential for the expression of MHC-II in thymic cortical epithelial cells [76]. Consequently, CIITA pIV KO mice show a severe defect in the positive selection of CD4+ T cells [76]. Thus, CIITA pIV is essential for the CIITA—and ensuing MHC-II—expression in cells of non-hematopoietic origin [21,74].

The role of CIITA pIII expression was addressed through pIII + IV KO mice [77]. These mice lack CIITA and MHC-II expression in all B cell subsets, which is not the case in pIV KO mice [77]. Furthermore, CIITA and MHC-II expression were absent in plasmacytoid DCs (pDCs) [77], which possibly are of lymphoid origin rather than myeloid cells such as conventional DCs [78]. In contrast to their murine counterparts, human activated T cells express CIITA and MHC-II. It was shown that this activity is mediated by CIITA pIII [79]. Taken together, this indicates that CIITA pIII is specific for cells of lymphoid origin [21,74].

CIITA pI was originally described as a DC-specific promoter [80]. It is also used for the constitutive and IFNγ-inducible expression of CIITA in macrophages and can thus be regarded as a myeloid-specific promoter [21,74]. Surprisingly, mice lacking CIITA pI and corresponding exon 1 had a relatively mild phenotype [81,82]. It was found that in pI KO animals, CIITA expression in DCs had shifted mainly to pIII. This suggested the involvement of distal regulatory elements in the expression of DC-specific CIITA and less tight insulation between pI and pIII compared to what had been found between pIII and pIV [81,82].

In addition to the proximal CIITA promoters, distal regulatory elements play a crucial role in CIITA expression. This has been best studied for IFNγ-induced expression of CIITA. Many distal regulatory elements involved in chromatin loops were identified from far-upstream to downstream of the CIITA-gene locus [83]. The chromatin-remodeling enzyme BRG1 was found to be crucial for the induction of histone modifications and the recruitment of STAT1, IRF1 and p300 [83]. BRG1-dependency is conferred on the CIITA locus by the polycomb repressive complex 2 (PRC2) [84]. More recently, it was shown that NFAT5 binding to an upstream distal regulatory element is essential for the IFNγ-dependent, pI-mediated CIITA expression in macrophages [85].

## 7. CIITA in Disease

In order to evade the immune response, several pathogens have evolved different mechanisms to suppress the antigen presentation pathway. Several viruses such as HIV prevent this presentation both at the level of MHC-II genes and molecules and at the level of CIITA gene expression [86,87,88,89]. Inhibition of MHC-II expression by varicella-zoster has also been observed [90,91]. Repression of CIITA by human cytomegalovirus (HCMV) and Epstein–Barr virus has also been shown [92,93,94,95]. Interference with the IFNγ pathway has been observed in infections with influenza viruses [96,97]. Herpes viruses have been shown to simultaneously inhibit CIITA and interfere with the transport of MHC-II molecules [98]. Kaposi’s sarcoma-associated herpes viruses have also been shown to inhibit transcription of CIITA via the viral protein vIRF3 and via SOCS3 [99,100]. *Toxoplasma gondii* downregulates CIITA and MHC-II in immune cells and neural cells by several different mechanisms [101,102,103]. Several species of *Mycobacterium* are capable of inhibiting CIITA via TLR-2 and chromatin modifications [104,105,106]. Chlamydia infection inhibits MHC-II expression via two different cell type-specific mechanisms, one chlamydial protease-like activity factor (CPAF)-independent and MyD88-dependent in bone marrow-derived macrophages and one CPAF-dependent in mouse embryonic fibroblasts [107,108].

Modulation of antigen presentation during viral infections is an important phenomenon, as inhibition of this pathway facilitates the spreading of the virus. HCMV has been shown to modulate several antiviral responses, including downregulation of MHC-I expression to inhibit CD8+ T cell activity [109,110]. However, certain viral effects on the CD4+ branch of immunity are not as well understood, mainly due to the absence of proper cellular models for the endogenous expression of MHC-II. Nevertheless, recent studies on the Kasumi-3 cell line were able to show that endogenous MHC-II downregulation by HCMV was mediated by inhibition of CIITA expression. This inhibition was also independent of previously known viral MHC-II repressor proteins that intervene in the IFNγ-induced expression, such as the tegument protein pp65, the viral IL-10 homolog, and two proteins coded from unique short (US) regions, US2 and US3, failed to downregulate MHC-II expression. Single gene knock-out studies failed to find a responsible gene in the US region, thus suggesting that the cause may be a complex interaction of several proteins in this region or that the responsible factor is coded elsewhere [95].

The Epstein–Barr virus is also known to evade the immune response by inhibiting CIITA. Expression of the immediate-early protein Zta in Raji cells has been shown to inhibit the expression of CIITA. This inhibition is mediated by Zta at the transcriptional level. Analysis of CIITA pIII revealed the existence of two Zta response elements (ZRE) in its sequence. The binding of Zta to these ZREs prevents transcription of CIITA and thus leads to inhibition of MHC-II expression [111].

## 8. CIITA-Dependent Regulation of Genes Other Than MHC-Genes

Children suffering from BLS never showed a phenotype that could not be explained by the absence of MHC-gene expression, indicating that CIITA and the three genes encoding RFX-subunits have an exquisite specificity for MHC genes. Given what we know about gene-regulation in general, it is, however, not astonishing that there are a limited number of other genes that appear to be regulated by CIITA, frequently in an RFX-dependent manner. A few genes possess well-defined W/SXY modules in their regulatory regions and are at least partially regulated by CIITA and RFX. Examples are the semaphorin receptor plexin-A1, RAB4B and butyrophilin-2A2 (BTN2A2) [112,113,114]. A certain number of genes have been reported to be negatively regulated by CIITA in IFNγ-induced cells. The best-studied example is probably collagen (COL1A2), where IFNγ-induced CIITA inhibits transcription in an RFX-dependent manner [115,116,117,118,119,120]. CIITA-mediated inhibition was dependent on the presence of the N-terminal acidic and P/S/T domains [116]. Recently, IFNγ-induced CIITA was shown to repress eNOS via the recruitment of the histone H3K9 trimethyl transferase SUV39H1 [121]. While it thus appears that CIITA-mediated inhibition is at least in part executed via the recruitment of co-repressors, it is not clear how the decision between activation and inhibition is made mechanistically. Some other genes have been proposed to be coregulated positively or negatively by CIITA (see discussion in [21,74]), but several of them could not be confirmed in vivo [122]. Taken together, it is apparent that CIITA is a highly dedicated and specific regulator of the MHC-II antigen presentation pathway.

## 9. Three’s a Crowd: NLRP3 as a T_H_2 Transcriptional Activator

Bruchard and colleagues reported that NLRP3, a well-known component of an inflammasome complex, can act as a transcriptional activator in CD4+ T_H_2 cells, contributing to the expression of T_H_2 cytokines, such as IL-4, IL-5, and IL-13 [123,124] (Figure 1). NLRP3 is normally cytoplasmic but was found to be localized in the nucleus of T_H_2 cells [123] (Table 1). Activation of the Il4 promoter occurred through interaction with IRF4. Similar results have recently been found in macrophages [125]. NLRP3 carries a pyrin domain in its N-terminus; however, the mechanism by which NLRP3 activates transcription was not investigated [123]. These results indicate that a third NLR protein can act as a transcriptional activator and raise the question of whether the same may apply to yet other members of this versatile protein family.

## 10. CIITA and Cancer

### 10.1. CIITA and Immune Evasion in Cancer

Given its function as a transcription factor regulating the expression of MHC-II genes, CIITA has been shown to play important roles in different pathologies. One of the most prominent examples of cancers where CIITA inactivation participates in tumor proliferation is the primary mediastinal large B-cell lymphoma (PMLBCL). This cancer is an aggressive form of non-Hodgkin’s lymphoma that often originates in the mediastinum, probably of thymic medullary B-cell origin [126]. These tumors present frequent genomic alterations in CIITA, comprising structural genomic rearrangements, missense, nonsense, and frameshift mutations found in 53% of primary tumor biopsies and PMBCL-derived cell lines [127]. Such alterations were also observed in an analysis of public data sets of whole transcriptome sequencing (RNA-seq) from three PMLBCL-derived cell lines: Karpas1106P, MedB-1, and U2940 [128]. Characterization of the CIITA-mutations in MedB-1 revealed two missense mutations (in trans) causing amino acid exchange in the acidic domain and the NACHT domain. Mutations in Karpas1106P presented two important deletions in both chromosomes; in one case, CIITA and the downstream SOCS1 gene are completely eliminated, while the other one results in disruption of CIITA. The cell line U2940 also presents an important deletion in one allele that generates a fusion of the upstream NUBP1 gene and CIITA, producing a fused transcript; the second allele contains a point mutation that generates a stop codon. In all cases, these modifications prevented the expression of the MHC-II genes and presumably helped the immune escape of the tumor [127]. These genetic modifications are so prevalent that their gene expression profile (GEP) can be used as a pattern for the identification of diffuse large B-cell lymphomas (DLBCL) without mediastinal participation that shows PMLBCL characteristics [129]. However, the immune escape phenomenon by PMBL is not only due to the inactivation of CIITA, as an integrative genomic analysis identified mutations in the Janus kinase–signal transducer and activator of transcription (JAK-STAT) and the nuclear factor κB (NFκB) pathways as concomitant factors participating to the immune escape [130].

Similar observations were made in cutaneous DLBCL. Analysis of 37 samples revealed that mutations that inhibit antigen presentation were second only to alterations in the NFκB pathway. Predictably, most of these mutations were present in B2M, CIITA and HLA genes [131]. Similar types of alterations have also been identified in a group of 20 patients suffering from primary cutaneous diffuse large B-cell lymphoma leg-type (PCLBCL-LT). Next-generation sequencing using a lymphoma panel for diffuse large B-cell lymphomas revealed frequent genetic losses in CIITA, as in previous cases, this loss may participate in the immune escape [132].

An important factor regulating the MHC-II expression in some blood cancers is Forkhead box P1 (FOXP1) [133]. This transcription factor has been observed as a poor prognosis marker in DLBCLs. Using microarray analysis of siRNA FOXP1-silenced DLBCL cell lines, differential regulation of MHC-II expression was identified between DLBCL expressing wild-type FOXP1 and activated-B-cell-like DLBCL, which expresses short isoforms of FOXP1. The cause of the downregulation of MHC-II was suggested to be FOXP1 acting as a novel negative regulator of CIITA target genes; thus, suppression of FOXP1 could represent a therapeutic target for the treatment of DLBCL [133]. Another factor involved in the expression of CIITA in cancer has been identified in pediatric glioblastomas. RACK7, a receptor for activated C-kinase, acts as an inhibitor of CIITA. RACK 7 recognizes a point mutation on histone H3.3 (H3.3G34R), often found in this type of tumor. Binding by RACK7 modifies the chromatin environment and suppresses the expression of CIITA, and thus limits the immunogenicity of the tumor [134].

Chronic myeloid leukemia (CML) is another type of blood cancer presenting CIITA aberrations important for the prognosis of the condition. It has been reported that MHC-II and CIITA are greatly downregulated in CML cases; however, the expression was increased following stimulation by IFNγ, thus proving that the MHC-II pathways remained functional [135]. Interestingly, expression of MHC-II and CIITA was significantly increased by inhibition of JAK1/2 via ruxolitinib. These results suggest that cytokine induction of the JAK-mediated pathway antagonizes MHC-II expression, thus presenting the opportunity for new therapeutic targets for the treatment of CML [135].

A strong downregulation of CIITA has been reported in blasts from acute myeloid leukemia (AML) relapse cases following allogeneic transplantation of hematopoietic cells. Inhibition of CIITA expression was concomitant with the upregulation of inhibitory checkpoint molecules that prevented T-cell recognition of AML cells and facilitated relapse [136].

Some cancers with viral infections have been shown to present upregulated expression of MHC-II. For example, Epstein–Barr virus-associated gastric adenocarcinomas (EBVaGC) presented a better outcome than EBV-negative cancers of the same type. The effect is suggested to be a consequence of upregulation of MHC-II expression that is concomitant with the expression of foreign viral antigens. Using RNA-seq data from 400 gastric cancer patients, the upregulation of all MHC-II involved genes, including CIITA and RFX5, was observed compared to normal tissues or other types of gastric cancers [137]. Expression of these genes was unexpected, as EBV infection induces expression of the viral protein BZLF1 (ZEBRA, Zta), which represses transcription of CIITA [111,138]. While expression of the viral protein was still found in many of these cancers, upregulation of MHC-II was correlated with higher levels of intratumoral IFNγ. These results suggested an APC function of cancer cells, increasing the immunogenicity of the tumor microenvironment and thus helping cancer elimination [137].

Another example of cancers where viral modulation of the MHC-II expression plays an important role is head and neck squamous cell carcinomas (HNSCC) [139]. A subset of these cancers is induced by high-risk human papillomaviruses (HPV) infections. Analysis of more than 500 RNA-seq data sets from HNSCC patients allowed the identification of the impact of HPV infections on the MHC-II expression and regulation. Upregulation of all genes involved in MHC-II antigen presentation roles was observed in HPV-positive cases compared to HPV-negative controls; among these genes were CIITA and RFX5 [139]. Similar to the observations of Ghasemi and colleagues, a viral infection of tumoral bodies was correlated with higher levels of IFNγ. This case also suggests an APC role for the cancer cells that participate in tumor elimination [137,139].

### 10.2. Generation of Cancer Vaccines via CIITA and NLRC5

The evident problem presented by the immune escape of tumors via suppression of antigen presentation has prompted some scientists to try to increase the immunogenicity of the cancer cells by artificially increasing the expression of the antigen-presenting pathways via the ectopic expression of CIITA or NLRC5.

The direct role of cancer cells as APCs has been investigated using a model of transgenic mice, the CD11c.DTR C57BL/6 mice (H-2^b^), where the dendritic cells (DC) can be functionally deleted via diphtheria toxin [140]. In order to eliminate other potential professional APCs, macrophages were also eliminated via the administration of liposome clodronate. In these mice, the H-2^d^ cells (epithelial cancers) were stably transfected with a plasmid carrying CIITA. Increased immunogenicity by the MHC-II expressing cells was able to successfully prime CD4+ T-cells and induce an antitumoral immune response in the absence of natural APCs [140]. From these observations, it has been proposed that antigens presented by MHC-II molecules and the induction of a T helper response could present a promising option to elicit an antitumor response that may even be more efficient than stimulating the MHC-I pathway [141].

Several studies have shown that artificial overexpression of CIITA, and thus of MHC-II, in cancer cells leads to increased antitumoral response. It has been shown that this expression significantly alters the tumor microenvironment by replacing a low population of tumor-infiltrating macrophages and neutrophils with activated CD4+ and CD8+ T-cells [141]. Using high MHC-II expressing cells, it has been possible to purify and isolate MHC-II bound peptides [142]. Application of this protocol to human hepatocarcinoma for the isolation of specific tumor peptides from both MHC-I and MHC-II pathways allowed the creation of the first multi-epitope, multi-target, and multi-allele vaccine targeting activation of CD4+ and CD8+ T cells. This vaccine was undergoing phase I/II clinical trials as of 2019, results from these trials should soon provide insights into the efficiency and safety of this method [141].

An important recent observation was made by Johnson and colleagues concerning the participation of MHC-II expression in anti-PD-1 therapies [143]. Two orthotopic immunocompetent murine models of non-small cell lung cancer, the cell line CMT167, which is sensitive to anti-PD1 therapy and the Lewis lung carcinoma (LLC), which is resistant, were used. RNA-seq analysis from in vivo samples of these cell lines revealed that sensitivity to the therapy was dependent on the CIITA-mediated expression of tumor-cell-specific MHC-II. Ectopic expression of CIITA in LLC rendered these cells MHC-II-positive and sensitized the tumors to anti-PD-1 therapy. Upregulation of CIITA was also associated with increased T-cell infiltration and a survival benefit in patient-derived lung adenocarcinomas [143].

Similarly, targeting the activation of the MHC-I pathways also has been explored. Activation of NLRC5 induces stimulation of the antigen processing machinery (APM) and expression of MHC-I molecules [38,39,40,144]. Importantly, it has been shown that inhibition of the expression of NLRC5 induced by promoter methylation, loss of copy number and somatic mutations induces strong downregulation of MHC-I, and the level of MHC-I expression is an important marker for the prognosis of the majority of solid cancers [145,146]. This suggested that ectopic expression of NLRC5 may be of therapeutic benefit. Stable transfection of NLRC5 into the murine melanoma cell line B16-F10 resulted in the upregulation of MHC-I molecules, TAP1, LMP2 and LMP7 (large multifunctional proteasome 2 and 7) [147]. This activation of the general APM leads to an efficient presentation of the tumoral antigen gp100 and subsequent activation of CD8+ T-cells specific for gp100. Injection of B16-F10 expressing NLRC5 (B16-5) in murine models showed efficient activation of the antitumoral immune response dependent on MHC-I expression. This immunity was translated into the reduced formation of tumor foci in the lungs and skin. Very interestingly, injection of irradiated B16-5 cells led to immunization against the parental cell line B16, which expresses very low levels of MHC-I molecules [147]. These results show the importance of both antigen-presenting pathways, not only in the antitumoral immunity but as possible targets for therapeutic treatments.

## 11. CIITA and Viral Inhibition

### 11.1. CIITA as a Viral Restriction Factor

An activity of CIITA that goes beyond its transcriptional regulatory function is its ability to act as an antiviral restriction factor (RF). Viral RFs are a relatively recent discovery and are part of a new branch of the immune system known as intrinsic immunity [148]. This mechanism acts by restricting viral replication and assembly of new viral particles and is mediated by proteins often expressed in a constitutive manner; however, some can also be induced by a viral infection. The most significant activity of restriction factors, when compared to other antiviral responses, is their ability to act immediately, thus being important factors in diminishing the effectiveness of the infection in early stages [148]. CIITA has been observed to behave as a restriction factor for human T cell leukemia virus-1 (HTLV-1), Human T cell leukemia virus-2 (HTLV-2) and human immunodeficiency virus-1 (HIV-1) [149,150,151]. CIITA was first shown as an RF against HIV-1 in T cells, where it competes for binding to cyclin-T1 (CyT1), a subunit of the *P*-TEFb complex, with the viral transactivator Tat [86,87]. Further studies on myeloid cells, another target of HIV-1, were performed in the promonocytic cell line U937, using mutants that allowed infection (U937 Plus) or not (U937 Minus) [152]. Comparing the differential expression of CIITA in these cell lines, these studies presented evidence that CIITA was able to suppress replication of HIV-1 by inhibiting transactivation of Tat, thus positioning CIITA as a general RF in myeloid cells as well as T cells [152,153]. This antiviral activity was observed to be independent of the activity of tripartite motif 22 (TRIM22), another RF that was identified before CIITA as an inhibitor of HIV-1 transcription [154]. Nevertheless, evidence suggests that TRIM22 and CIITA cooperate in their RF activity. This cooperation is mediated by the assembly of a nuclear body, a protein complex containing CIITA, TRIM22 and the promyelocytic leukemia protein (PML) [155]. The nuclear bodies then compete for and capture CyT1, thus preventing the activation of Tat. This mechanism constitutes the first example of synergistic activity in intrinsic immunity [155].

The activity of CIITA as an RF for HTLV-1 is mediated by a direct interaction between CIITA and the viral protein Tax-1. This binding prevents the interaction of Tax-1 with its coactivators p300/CBP-associated factor (PCAF), the cyclic AMP-responsive element-binding protein (CREB), and the activating transcription factor-1 (ATF1), which are required for the proper activation of HTLV-1 [150]. This mechanism also has been shown to participate in CIITA’s inhibition of HTLV-1 induced oncogenic transformation of T cells. This transformation is induced by persistent activation of the NF-κB pathway mediated by Tax-1. Interestingly, CIITA has been observed to inhibit the NF-κB pathway via several mechanisms; the interaction between CIITA and Tax-1 in the cytoplasm prevents activation and nuclear transport of p50/RelA/IκB complexes, while nuclear bodies containing CIITA bind Tax-1/RelA and block activation of NF-κB controlled genes [153]. As mentioned before, CIITA is also able to act as an RF for HTLV-2, similar to the mechanism observed for HTLV-1. CIITA interacts directly and inhibits the activity of the Tax-2 transactivator. However, the domains that interact between CIITA and both Tax proteins are different, but overlapping in each case; for Tax-1, CIITA interacts via two adjacent regions comprising amino acids 1 to 252 and 253 to 410, while the interaction with Tax-2 is mediated by the N-terminal domain between amino acids 1 to 321 and seems to act in cooperation with NFY [156].

### 11.2. CIITA as an Inhibitor of Ebola and Corona Viruses

Recently, Bruchez and colleagues showed that CIITA inhibited the Ebola virus and coronaviruses, although CIITA was acting in this instance as a conventional MHC-II gene activator and not as a viral restriction factor [157]. With the objective of identifying pathways that participate in resistance mechanisms to viral infections, a transposon-mutagenesis forward screen in U2OS cells was applied. Transposon-modified cellular libraries were treated with vesicular stomatitis virus expressing the Ebola glycoprotein (EboGP-VSV), and cells surviving the infection were expanded and analyzed by next-generation sequencing of the transposon insertion sites. In addition to the Ebola virus receptor NPC1, only CIITA was identified repeatedly as a transposon target. Transposon insertion resulted in the upregulation of CIITA, which increased the cellular resistance to EboGP-VSV between 100 and 1000-fold. Further experiments suggested that CIITA inhibits viral entry by blocking fusion in the endosomes. Given that CIITA acts as a transcription factor, genes controlled by CIITA were analyzed. Among these, only the isoform p41 of CD74, the invariant chain of the MHC-II molecules, was able to induce viral resistance. Expression of CD74 fragments pinpointed the resistance ability to the thyroglobulin domain, whose presence in the endosome inhibits the activity of cathepsins, which are needed for the processing of the EboGP and subsequent viral fusion. Similar glycoprotein processing is needed for the insertion of other viruses, such as coronaviruses. Expression of p41-CD74 in U2OS and Vero cells was able to block entry of pseudotyped viruses expressing the S protein from SARS-CoV-2 and WIV1-CoV, thus confirming that p41-CD74 inhibited processing of the viral protein. These experiments were important for the identification of new mechanisms dependent on CIITA that are astonishingly separated from the antigen-presenting pathways. These experiments also suggest that there may be novel and important functionalities for CIITA left to identify [157].

## 12. Conclusions

In this review, we have attempted to give an overview of the biology of CIITA, the master regulator of MHC-II expression. CIITA and its closest homolog NLRC5 are highly dedicated regulators of MHC-gene expression and are therefore of crucial importance for the regulation of the cellular immune response. Differential expression of MHC-II gene expression is mediated by the differential expression of CIITA, which is controlled by three independent promoters. As could be expected, CIITA is a major target for cancer immune escape mechanisms, and ectopic expression of CIITA and NLRC5 present promising avenues in cancer vaccination strategies. Finally, CIITA acts as a viral inhibitor, both as a viral restriction factor and through its role as an MHC-II gene regulator, demonstrating its crucial role for the immune response.

## Figures and Tables

**Table 1:** Features of the NLR proteins CIITA, NLRC5 and NLRP3.

	CIITA	NLRC5	NLRP3
Aliases	NLRA; C2TA; MHC2TA	NOD27; CLR16.1	CIAS1; PYPAF1; Cryopyrin; CLR1.1; NALP3
Main functions	MHC-II gene transcription	MHC-I gene transcription; diverse roles in innate immunity	Inflammasome; transcriptional regulator of T_H_2 differentiation
Length (human)	FI: 1207 aa ^a^ FIII: 1130 aa FIV: 1106 aa	1886 aa	1036 aa
Subcellular localization	Nuclear and cytoplasmic Lb ^b^: nuclear	Cytoplasmic Lb: nuclear	Nuclear and cytoplasmic (in T_H_2 cells)
Interaction partners ^c^	RFX5; RFX-ANK; NF-YB; NF-YC; CREB; Bob-1; p300; p400; PCAF; CBP; BRG1; CARM1; TFIID; TFIIB; *P*-TEFb; Sug1; SIRT1; ZXDA; ZXDC	RFX-ANK	IRF4

Footnotes: ^a^: amino acids; ^b^: leptomycin B treatment; ^c^: only considering transcriptional regulatory functions.
